# Genetic Relationship Among the Kazakh People Based on Y-STR Markers Reveals Evidence of Genetic Variation Among Tribes and Zhuz

**DOI:** 10.3389/fgene.2021.801295

**Published:** 2022-01-07

**Authors:** Elmira Khussainova, Ilya Kisselev, Olzhas Iksan, Bakhytzhan Bekmanov, Liliya Skvortsova, Alexander Garshin, Elena Kuzovleva, Zhassulan Zhaniyazov, Gulnur Zhunussova, Lyazzat Musralina, Nurzhibek Kahbatkyzy, Almira Amirgaliyeva, Mamura Begmanova, Akerke Seisenbayeva, Kira Bespalova, Anastasia Perfilyeva, Gulnar Abylkassymova, Aldiyar Farkhatuly, Sara V. Good, Leyla Djansugurova

**Affiliations:** ^1^ Institute of Genetics and Physiology, Almaty, Kazakhstan; ^2^ The University of Winnipeg, Winnipeg, MB, Canada; ^3^ Al-Farabi Kazakh National University, Almaty, Kazakhstan; ^4^ Oskemen Bilim-Innovation Lyceum, Ust’-Kamenogorsk, Kazakhstan

**Keywords:** Y-chromosome, Y-STR, haplotypes, haplogroups, Kazakhstan, MDS plot, Kazakh tribes

## Abstract

Ethnogenesis of Kazakhs took place in Central Asia, a region of high genetic and cultural diversity. Even though archaeological and historical studies have shed some light on the formation of modern Kazakhs, the process of establishment of hierarchical socioeconomic structure in the Steppe remains contentious. In this study, we analyzed haplotype variation at 15 Y-chromosomal short-tandem-repeats obtained from 1171 individuals from 24 tribes representing the three socio-territorial subdivisions (Senior, Middle and Junior zhuz) in Kazakhstan to comprehensively characterize the patrilineal genetic architecture of the Kazakh Steppe. In total, 577 distinct haplotypes were identified belonging to one of 20 haplogroups; 16 predominant haplogroups were confirmed by SNP-genotyping. The haplogroup distribution was skewed towards C2-M217, present in all tribes at a global frequency of 51.9%. Despite signatures of spatial differences in haplotype frequencies, a Mantel test failed to detect a statistically significant correlation between genetic and geographic distance between individuals. An analysis of molecular variance found that ∼8.9% of the genetic variance among individuals was attributable to differences among zhuzes and ∼20% to differences among tribes within zhuzes. The STRUCTURE analysis of the 1164 individuals indicated the presence of 20 ancestral groups and a complex three-subclade organization of the C2-M217 haplogroup in Kazakhs, a result supported by the multidimensional scaling analysis. Additionally, while the majority of the haplotypes and tribes overlapped, a distinct cluster of the O2 haplogroup, mostly of the Naiman tribe, was observed. Thus, firstly, our analysis indicated that the majority of Kazakh tribes share deep heterogeneous patrilineal ancestries, while a smaller fraction of them are descendants of a founder paternal ancestor. Secondly, we observed a high frequency of the C2-M217 haplogroups along the southern border of Kazakhstan, broadly corresponding to both the path of the Mongolian invasion and the ancient Silk Road. Interestingly, we detected three subclades of the C2-M217 haplogroup that broadly exhibits zhuz-specific clustering. Further study of Kazakh haplotypes variation within a Central Asian context is required to untwist this complex process of ethnogenesis.

## Introduction

**FIGURE 1:**
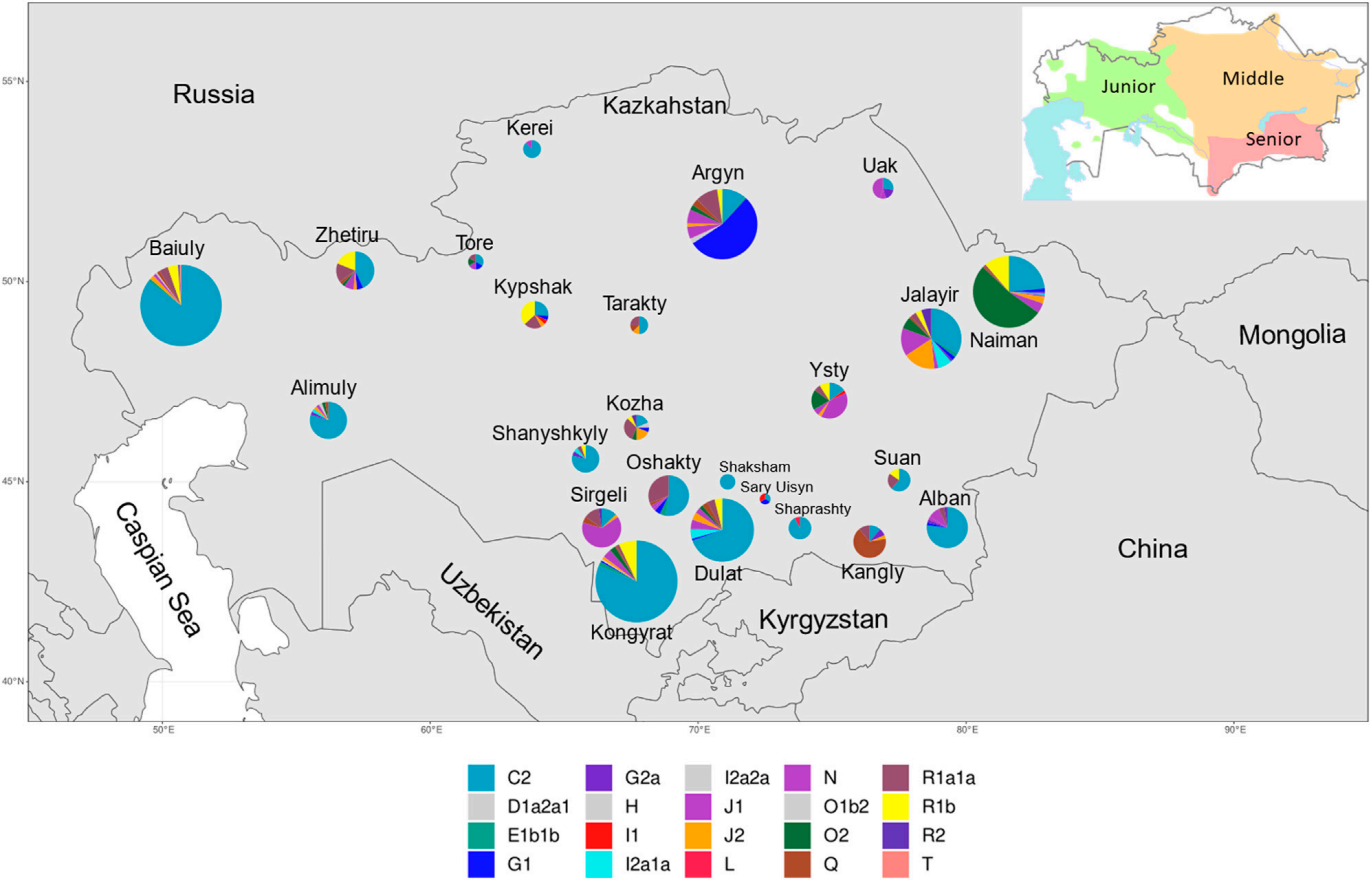
Frequency of Y-STR haplogroups by approximate geographic center of 22 Kazakh tribes, plus the Kozha and Tore in Kazakhstan. Size of pie chart is proportional to the total number of individuals sampled. The relative proportion of each haplogroup (*see* legend for colouration) is depicted in each pie chart. The smaller map in the top right corner shows approximate territories of Kazakh zhuzes in early 20th century. Reproduced from Wassily 2014.

Central Asia is a region populated by a wide range of ethnicities and characterized by heterogeneous economic and linguistic landscapes. Being located along the Silk Road, Central Asian populations have been genetically and culturally influenced by a millennia-long interplay between East and West that underpins its highly diverse genetic landscape. Genetic studies of Bronze Age (3100–1300 BC) remains from Central Asia show substantial temporal changes in the genetic composition of populations, indicating extensive migrations and west-to-east expansions of sedentary herders from the western steppe that formed a homogeneous gene pool by the end of the second millennium BCE (Allentoft et al., 2015; Narasimhan et al., 2019; Lalueza-Fox et al., 2004). In the Iron Age (1300–900 BC), nomadic pastoralists spread through the Eurasian steppe, dispersing the Scythian culture. Analysis of ancient DNA from Sakas and Sarmatians burials, belonging to the Scythian culture, demonstrates an increase of Iranian and eastern Eurasian genetic influx in southern and eastern samples, respectively (Gnecchi-Ruscone et al., 2021; Unterländer et al., 2017). During the first millennium CE, multiple confederations and empires were formed on the territory of modern Kazakhstan that were associated with substantial gene flow. For instance, the male-biased westward expansion of the Xiongnu nomads from the eastern steppe led to significant admixture of east Eurasian lineages into Central Sakas and displacement of the Indo-European Kangju and Wusun people (Damgaard et al., 2018). Subsequently, diverse Turkic nomadic states formed and blended into each other, resulting in gene flows between heterogeneous populations of the former Hunnic empire (Damgaard et al., 2018; Gnecchi-Ruscone et al., 2021). Following the Mongol invasion of the territory in 1211, the Golden Horde was established in the 13th century, that underwent series of fragmentation in the following centuries, resulting in the establishment of the Kazakh Khanate (1465–1847). During this time, nomadic tribes of different origins lived throughout the territory of present-day Kazakhstan, and eventually they were organized into three socio-territorial groups (zhuzes) based largely on geographical origin: Senior zhuz, Middle zhuz, and Junior zhuz (Figure 1) (Akishev et al., 1996).

The nomadic society of the Kazakh Steppe was organized based on a hierarchical patrilineal clan system of genealogical lineages. Individuals of the same genealogical lineage claim to share a common ancestor, and multiple genealogical lineages combine into clans that, collectively, form tribes. The 12 tribes of the Senior zhuz primarily occupied Southern and South-Eastern Kazakhstan, the seven tribes of the Middle zhuz reside in Eastern, Northern and Central Kazakhstan, while the three tribes in the Junior zhuz traditionally lived in Western Kazakhstan (Figure 1). Some of the steppe clans were not affiliated to the zhuzes, notably the clergy (Kozha and Sunak) and aristocracy (Tore). Representatives of the Kozha and Sunak clans link their ancestry to Islamic missionaries who originated from paternal-line relatives of the Prophet Muhammad. Tore people claim to be direct descendants of Genghis Khan. In contrast to sedentary farmer populations of Central Asia, Kazakhs have practiced exogamous marriages: a partner must be chosen from a different clan, and women integrate into the clan of their husband.

Despite the globalization of the last centuries and the move to a sedentary lifestyle, the tribal-clan structure of the Kazakh people has persisted, and many modern Kazakhs know the tribal affiliation and history of their clan. Being a patrilineal custom, the transgenerational transmission of tribal-clan affiliation resembles inheritance of the non-recombining part of Y chromosome, even though the former is a social entity. The analysis of genetic markers of the Y chromosome has been successfully employed in many studies of human populations to reconstruct migration routes; the combination of extended Y-haplotypes in patrilocal communities with genealogical data can enhance our understanding of the fine-scale demographic dynamics of a population (Wells et al., 2001).

To date, a multitude of studies has employed genetic markers to investigate the genetic diversity and differentiation of the Kazakh population in global (Wells et al., 2001; Underhill et al., 2010; Underhill et al., 2015; Unterländer et al., 2017), regional (Karafet et al., 2002; Lalueza-Fox et al., 2004; M.; Zhabagin et al., 2017) and local (Gokcumen et al., 2008; Balmukhanov et al., 2013; Tarlykov et al., 2013; Wen et al., 2020; M.; Zhabagin et al., 2021) contexts. The accumulated data provide preliminary insights into the demographic history of Kazakhs. For instance, Central Asian populations possess high levels of mtDNA and Y chromosomal haplotype diversity (Underhill et al., 1997; Comas et al., 2004), although paternal genetic markers are less polymorphic than maternal ones (Gokcumen et al., 2008; Tarlykov et al., 2013; Shan et al., 2014a). An earlier study also indicated potential discrepancies between the present-day geographic distribution of the Kazakh tribes and their ancestral relationships to neighboring populations (Tarlykov et al., 2013). Overall, however, prior studies have been hampered by one or more weaknesses such as small sample size, disregard for tribal affiliation, genealogical information, and/or insufficient geographical coverage. Here, we performed one of the largest study to date, *N* = 1,171 of Y-chromosomal haplotype diversity among all extant Kazakh tribes including the Tore and Kozha, with the primary goal of assessing the relationship of tribes and zhuz among the Kazakh people of modern-day Kazakhstan.

## Materials and Methods

*Samples and DNA extraction:* A total of 1171 Kazakh males were included in this study. Blood or saliva was sampled from unrelated males from all-known Kazakh clans throughout five geographic regions in Kazakhstan (Supplementary Tables S1, S2). Individual and ethnological information, such as ethnicity and tribal-clan affiliation were self-declared and collected using an approved interview form from all individuals for which blood/saliva samples were obtained. Individuals with admixed ethnicity in their paternal lineage were removed from the study. Written informed consent was obtained from all participants to perform genetic analyses. The study was approved by the local ethics committee for biological research at the National Center for Biotechnology. Genomic DNA was extracted from all samples using the QIAamp DNA Mini Kit (Qiagen, Germany) according to the manufacturer’s protocol and quantified spectrophotometrically (BioPhotometer Plus, Eppendorf, Germany) and fluorometrically (Qubit 2.0, Thermo Fisher Scientific, United States).

*Y-STR genotyping:* Samples were genotyped with the AmpFLSTR Y-filer PCR Amplification Kit (Thermo Fisher Scientific, United States) generating STR profiles at 17-loci (DYS19, DYS389I, DYS389II, DYS390, DYS391, DYS392, DYS393, DYS385a, DYS385b, DYS438, DYS439, DYS437, DYS448, DYS458, DYS456, DYS635, and Y-GATA-H4) (Supplementary Table S2). Fragments were separated and visualized on the ABI PRISM 310 Genetic Analyzer (Applied Biosystems, United States) and alleles called using GeneMapper IDX V1.4 (Applied Biosystems, United States). Due to difficulties in correctly assigning alleles to the duplicated DYS385a/b loci, these loci were removed and the haplotype of all individuals at 15 loci were used in further analyses. The raw data were submitted to the Y-Chromosome Haplotype Reference Database (YHRD) and is under the accession number YA004686.

*Haplogroup prediction and Y-SNP genotyping:* Y-haplogroups were predicted using the online Y-DNA Haplogroup Predictors NevGen (http://www.nevgen.org) as well as Whit-Athey’s (http://www.hprg.com). Putative Y-haplogroups for 16 of the 20 predicted haplogroups were then definitively determined through the analysis of 16 Y-SNPs, genotyped by RFLP or allele specific PCR using nine primers designed from previous studies and seven primer pairs designed for this study (Supplementary Table S3). In Y-STR haplotypes confirmed by Y-SNP analyses were confirmed for 1164 men (Supplementary Table S2). The nomenclature of Karafet et al., 2008, Underhill et al., 2010, and Myres et al., 2011 was used for SNP-analyses as they incorporate the latest information from the International Society of Genetic Genealogy (ISOGG) regarding Y-haplogroup confirmation (https://isogg.org/tree/index.html).

## Data Analyses

*Haplogroups:* To visualize the clustering of Y-haplogroups in the region, the Y-haplogroups of all individuals were plotted with respect to their birthplace and sampling location (Supplementary Figures S1, S2, respectively). Haplogroup frequencies were calculated by direct counting and the frequency of Y-haplogroups by tribe calculated and plotted with respect to the approximate geographic center of the tribes.

*Haplotypes:* The frequency of alleles (repeat lengths) for each Y-STR marker was calculated by direct counting, and the genetic diversity (GD) of single-markers was calculated using Nei’s formula GD = n(1-Σp_i_^2^)/(n-1), where P_i_ is the relative frequency of the i^th^ allele and n is the sample size (Nei, 1987). The number of unique haplotypes and their frequencies were obtained with the help of the R package Pegas (Paradis, 2010). Haplotype diversity was calculated in an analogous way to GD except by replacing the allele frequencies (Pi) by the relative frequencies of each haplotype. Haplotype discrimination capacity (DC) was calculated as the ratio of unique haplotypes in the sample (i.e. (n haplotypes)/N) ∗100).

*Genetic distance and differentiation among tribes:* The pairwise genetic distances between individuals in each of the 24 tribes was estimated using Weir and Cockerham pairwise FST for haploid data (Weir and Cockerham 1984). An analysis of molecular variance was performed to assess the components of molecular variance as explained by both tribe (model: ∼tribe), and tribe nested in zhuz (∼zhuz/tribe). The significance of the covariance components was assessed using a permutation test following Excoffier (Excoffier et al., 1992). A Mantel test was performed to test for a linear relationship between the Euclidean geographic distance between all pairs of individuals’ birthplace and the corresponding genetic distance of their Y-haplotypes estimated by Edward’s distance, and the significance of the regression was tested by a randomization procedure as implemented in the R-package Ade4 (v 1.7–15).

*Structure and Principal Coordinate Analysis:* An analysis of the inferred number of ancestral Y-STR groups, K was performed using the program Structure 2.3.4 (Pritchard et al., 2000). Given the nearly complete linkage and haploid nature of the Y-STR, we assessed the haplotype structure within haplogroups and tribes using a “no admixture” model, which assumes that each individual originate from one of the K ancestral groups. The structure analyses was performed on 1163 individuals (all of those that were SNP-genotyped and excluding haplogroup T present in a single individual) using a person’s tribal affiliation and haplogroup as priors. To assess the best estimate of K, the number of ancestral Y-STR groups in the total population, simulations were carried out for K = 1 to 25 with three replicates for each value of K. To select the best estimate of K, the second-order rate of change of the likelihood function with respect to K was estimated, using the program Structure Harvester (Earl and vonHoldt 2012), and the value of K with the highest likelihood selected. We also examined the inferred posterior probability that individual i is from the *k*th group assuming a prior probability of 1/K. To further explore the relationship among tribes, a principal coordinate analysis (PcoA aka, multidimensional scaling, MDS) was performed by using the pairwise genetic distance between individuals based on Meirmans PT, as above, performing the PcoA and then extracting the first two dimensions for visualization with the help of the R package Ape (Paradis et al., 2004). The individual coordinates of all 1171 individuals were plotted with respect to *1*) Y-haplogroups and *2*) the tribal affiliations of individuals.

## Results

*Y-chromosome haplogroups in Kazakh population*: 1171 Kazakh males were included in the study: 433 total individuals representing the 12 tribes in the Senior zhuz, 475 from seven tribes in the Middle zhuz, 241 from the Junior zhuz, and 22 samples from the Kozha (*n* = 16) and Tore (*n* = 6) tribes (Supplementary Table S1). Although the analysis performed here is based on the highest hierarchical affiliation of a person to their tribe/zhuz, all but 142 individuals reported their family lineage/clan and this data could be used in future analyses (given in Supplementary Table S2). All individuals included in the study were living in Kazakhstan at the time of the study, 61 were born outside of Kazakhstan (Supplementary Table S2, Supplementary Figure S1 for map of birth locations and Supplementary Figure S2 for sampling locations). Gene diversity of the single locus markers for each of the 15 Y-STR’s ranged from 0.3305 (DYS438) to 0.7699 (DYS635) (Supplementary Table S4). The locus with the highest diversity, DYS635, harbored eight allelic classes, while the least diverse loci DYS393, DYS391 and DYS437 each had five alleles, with 129 alleles scored at all 15 loci (Supplementary Table S4). Haplotypes were submitted to two online Y-DNA haplogroup predictors (NevGen and Whit-Athey’s) to assign a tentative haplogroup to each individual (given in Supplementary Table S2). Sixteen of the 20 predicted haplogroups were confirmed by SNP genotyping using primers obtained from the literature or developed for this study (Supplementary Table S3). Of the 1171 males for which a Y-haplotype was obtained, 1164 were assigned to one of 16 Y-haplogroups based on the combined Y-STR - SNP data, while seven individuals were assigned to one of four additional Y-haplogroups (D1a2a1, H, I2a2a, O1b2) based on the NevGen prediction alone (Supplementary Table S2). These seven individuals were included in some analyses (those based purely on haplotypes for which only the allele sizes/locus are used) but not all analyses; these four haplogroups require Y-SNP genotyping to be confirmed.

The most frequent haplogroup in the Kazakh population was C2-M217 (51.9% - 608 men). Haplogroup C2-M217 was present in all examined tribes and its frequency ranged from 11% in Kangly and Argyn to 100% in Shaksham (*n* = 6) (Figure 1 and Supplementary Table S5). Another important component of the Kazakh gene pool is represented by the haplogroup R (12.8%), which has three subclades: R1a1a-M17 (6.5%, 76 individuals), R1b-M343 (5.6%, 65 individuals) and R2-M479 (0.8%, nine individuals). The R1a1a-M17 haplogroup was observed in 18 of the 24 tribes, and was most frequent in the Kozha clan (31.3%, five individuals) and Oshakty tribe (31%, 13 individuals). Subclade R1b-M343 was found in 12 Kazakh tribes and had the highest frequency (36.8%, seven individuals) in the Kypshak tribe. Lastly, the subclade R2-M479 was observed in five tribes at low frequencies, and was most prevalent in the Kozha clan (6.3%, one person) and Jalayir tribe (5.4%, five individuals) (Figure 1 and Supplementary Table S5).

Haplogroups O (represented by O2-M122 and O1b2, the latter predicted by NevGen), G (G1-M285 and G2a-P15), N-M231, J (J1-M267 and J2-M172), and Q-M242 were observed at frequencies <10% across tribes, but were found at higher frequencies in one or a few tribes. For example, haplogroup O2-M122, had a frequency of 8.03% in the sampled population, but was found in 52.3% of the individuals from the Naiman tribe. Haplogroup G had a global frequency of 7.9%, with the majority (7.1%) belonging to subclade G1-M285 and 0.8% to subclade G2a-P15, but the frequency varied between tribes, and ∼54% of the males from the Argyn tribe (*n* = 126) harbored haplogroup G1-M285. The highest frequency of the haplogroup G2a-P15 was observed in the Uak tribe (18.2%, 11 individuals). The N-M231 haplogroup had a global frequency of ∼6.9%, but was prevalent in the Sirgeli (64.1% of 39 individuals) and Uak (45.5% of 11 individuals) tribes, Tore tribe (16.7% from six individuals) and Jalayir tribe (15.1% from 93 individuals). Haplogroup J, represented by J1-M267 and J2-M172, had a global frequency of ∼6.2%, being frequent in the Ysty tribe (J1-M267 (39.4%, 13 individuals) and J2-M172 (3%, one person)). Additionally, J2-M172 was also observed in the Kozha clan (18.8%, three individuals). Lastly, haplogroup Q had a low overall frequency of ∼3.1%, but was highly represented in the Kangly tribe (66.7%, 27 individuals), while its frequency was <5.5% in all other tribes (Supplementary Table S5). The other haplogroups show frequencies in the sampled population lower than 2% (Supplementary Figure S3).

The assigned Y-haplogroup of each individual was plotted with respect to the location of their birth and sampling location (Supplementary Figures S1, S2) and the frequency of the haplogroups by tribe was plotted with respect to the approximate geographic center of the territory occupied by a tribe in the past (Figure 1). Overlaying the Y-haplogroup assignments on the map of Kazakhstan with the approximate route of the Mongolian invaders who rampaged through Central Asia in the 13th century (Zerjal et al., 2003), shows the historical mark of this invasion since haplogroup C2 is more frequent in the southern and western portions of the country where the Mongols passed (Supplementary Figure S1). For example, tribes in the southern and western regions of Kazakhstan, such as the Alban, Kongyrat, Dulat, Baiuly and Alimuly, have frequencies of C2-M217 ≥ 70%, while tribes located in the center and northeast of the country, such as the Argyn, Uak, Naiman, Kangly, Ysty and Kypshak, have lower frequencies (<30% in most cases) (*see* Figure 1 and Supplementary Table S5).

*Haplotype Diversity:* From the 1171 individuals, 577 distinct haplotypes were found, of which 429 were observed once and the remaining 148 were observed between 2 and 51 times and 15 haplotypes observed ≥10 times (Supplementary Table S6). Overall, this resulted in a Y chromosome haplotype diversity of 0.9938 ± 0.0006, reflecting the deep paternal lineages of different origins in the sample. However, the discriminatory capacity of the samples based on the haplotype frequency distribution was 55.17%, reflecting the high frequency of a few haplotypes. Seven of the top nine most frequent haplotypes belonged to the C2 haplogroup (Supplementary Table S6). The two most common haplotypes (Ht1, Ht2, Supplementary Table S6) belonged to the C2 haplogroup and were both observed in 51 individuals while a third C2 haplotype (Ht3) was observed in 37 individuals. Ht1 was present in 38 individuals from the Baiuly tribe and six individuals from the Alimuly tribe (Supplementary Table S7) both of which belong to the Junior zhuz, located in western Kazakhstan (Figure 1). Ht2 was identified in 32 individuals in the Dulat tribe and 10 individuals in Alban, both belonging to the Senior zhuz in the southeastern region (Figure 1 and Supplementary Table S7). Finally, Ht3 was observed in 37 individuals from the Kongyrat, a member of the Middle zhuz (Figure 1 and Supplementary Table S7). The most frequent non-C2 haplotype (Ht4) belonged to haplogroup G1 and was found in 34 individuals, 26 of which were in the Argyn tribe belonging to the Middle zhuz, located in the center-north of the country (Figure 1 and Supplementary Table S7).

*Population Structure:* The analysis of molecular variance revealed that between ∼73% (Model: ∼tribe) and ∼71% (Model: ∼zhuz/tribe) of the variation in Y-STR diversity is attributable to variation within tribes. Variation in Y-STR diversity among zhuz, explained ∼9% of the variation in YSTR-diversity, a value that was lower than expected if the diversity was distributed randomly among the highest hierarchical level using a Monte Carlo permutation test, while the amount of variation within tribes was greater than expected (Table 1 and Supplementary Figure S4). Estimates of the fixation index ΦST, were significant and between 0.273 (model ∼tribe) and 0.2923 (model ∼ zhus/tribe), such that between 27.3 and 29.3% of the total variation is due to inter-tribal differentiation (Table 1). Pairwise Weir and Cockerham’s F_ST_ values between tribes varied between ∼0 (−0.0003, Baiuly vs Alimuly) and 0.19 (Sirgeli vs Shaksham) (Supplementary Table S8).

**TABLE 1:** Analysis of Molecular Variance (AMOVA) among Y-STR haplotypes of 1171 men in Kazakhstan using two models *1*) tribal affiliations (model: ∼tribe), and *2*) tribal affiliation nested in Zhus (model: ∼zhus/tribe).

Structure design	Source of variation	d.f.	Sum of squares	Variance components (sigma)	Percentage of variation	Permutation test (*α* = 0.01)
Model: ∼Tribe	Among tribes	23	2792.23	2.47	26.77	Less
	Within tribes	1147	7751.01	6.75	73.23	Greater
	Total	1170	10543.24	9.01	ΦST: 0.268	
Model: ∼Zhuz/Tribe	Among zhuzs	2	1103.96	0.844	8.91	Less
	Among tribes Within zhuzs	21	1688.27	1.86	19.68	Less
	Within tribes	1147	7751.01	6.75	71.40	Greater
	Total	1170	12353.99	11.125	Φ_SC_: 0.216	
					Φ_ST_: 0.286	
					Φ_CT_: 0.089	

The significance of the covariance components was tested using a Monte Carlo permutation test using an alpha = 0.01: variance components that were less than or greater than expected under the null (permuted) distribution are marked as < or > respectively.

*Mantel test:* Despite the apparent higher frequency of the C2-M217 haplogroup along the southern and western borders of Kazakhstan, tracking the Mongolian invasion (Supplementary Figure S1), there was not a significant correlation between the genetic and geographic distance among individuals based on a linear regression between all individuals pairwise. The regression line explained only 0.12% of the variation in the data and the permutation test failed to reject the hypothesis of no spatial structure (Supplementary Figure S5).

**FIGURE 2:**
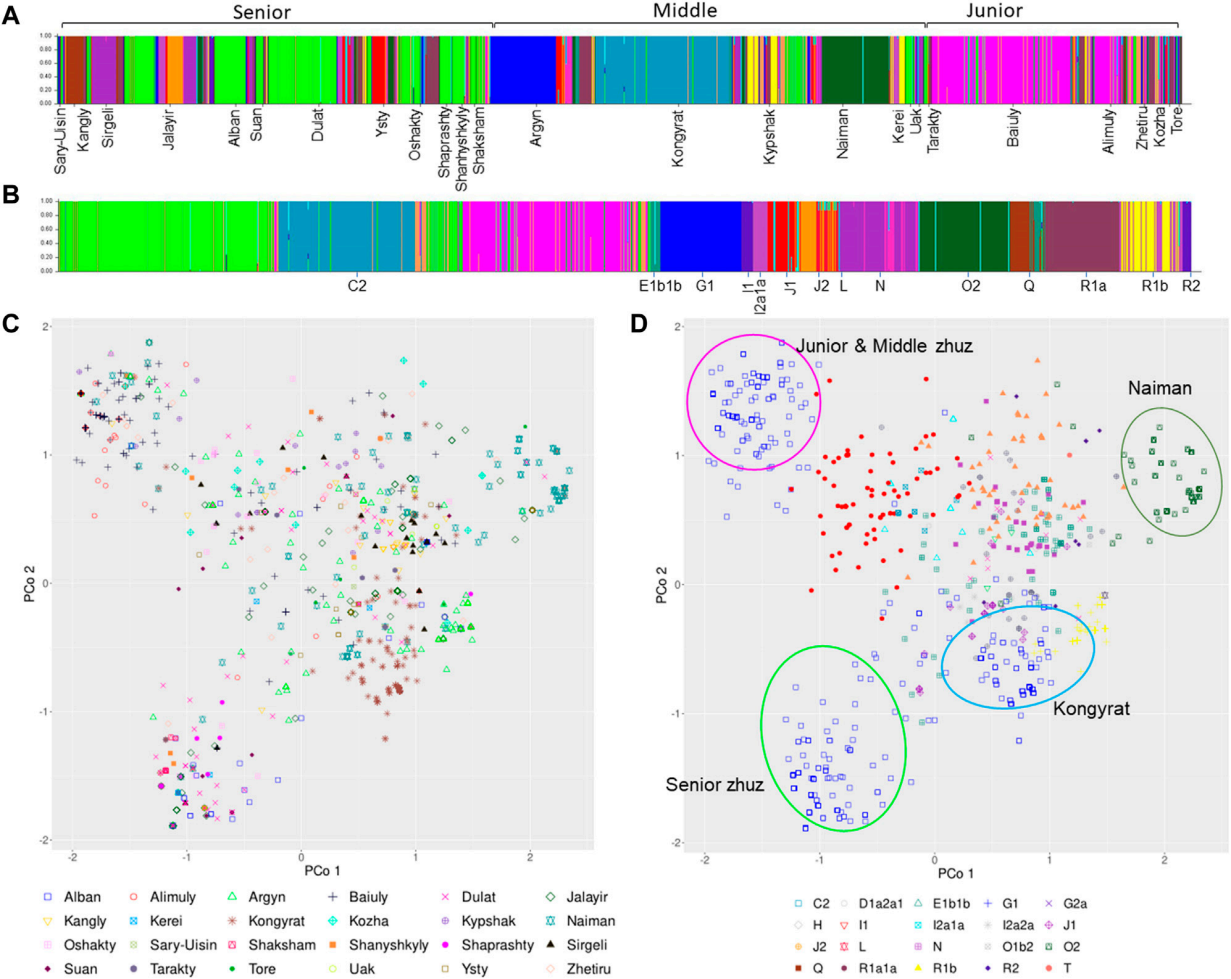
Posterior Probability that an individual is assigned to a specific tribe **(A)** or haplogroup **(B)** based on a no admixture model in the program Structure. Principal Coordinate Analysis (also known as Multidimensional Scaling Plot) showing the relationship among 1171 Kazakh men genotyped at 17 Y-STR’s by tribe **(C)** or and haplogroup **(D)**.

*Structure analyses*: Further analysis of the haplotype structure using the program Structure implementing a “no admixture” model and using both the tribe and haplogroup as priors, found that the best estimate of K was 20 (Supplementary Table S9). Visualization of the structure of the Y-STR’s by tribe (Figure 2A) and haplogroup (Figure 2B), confirms the presence of at least three subgroups within C2-M217: lime green (Senior zhuz), teal blue (Middle zhuz) and pink (Junior zhuz). Similarly, it identified the presence of some homogenous haplogroups (e.g. G1, O2 and R1a1a) that are strongly associated with some tribes (e.g. G1- Argyn, O2 – Naiman).

*MultiDimensional Scaling:* The principal coordinate analysis (PCoA) provided a closer examination of the relationship among A) tribes and B) haplogroups. This revealed that while many of the haplogroups fall in the middle of the coordinate with somewhat distinct clustering (Figure 2D), the haplogroups are diffusely distributed among tribes (Figure 2C). On the other hand, there are three distinct clusters of haplogroup C2-M217 that fall in the upper and lower left or middle bottom (Figure 2B), which broadly correspond to the three subgroups found in the Structure analyses. Haplogroup C2-M217 cluster 1 is found within numerous tribes in the Junior and Middle zhuz, notably the Baiuly, Argyn, Alimuly and Kypshak and corresponds to the pink haplotypes in the Structure analyses (Figures 2B,D). On the other hand, the second C2-M217 haplogroup is found among diverse members of the Senior zhuz, including the Alban, Shaprashty, Oshakty, Dulat and Suan, all found in south-east Kazakhstan and corresponds to the lime-green Structure haplotypes (Figures 2B,D). Lastly, the third cluster of C2 haplogroups is found predominantly among members of the Kongyrat tribe corresponding to the teal blue Structure haplotype (Figures 2B,D). Other distinct clusters in the MDS analyses pertain to haplotype O2 (upper right, Figure 2D), which is most frequent in the Naiman tribe, but is also found in the Jalayir tribe (Figure 2C) and for haplotype G1 (brown crosses middle right, Figure 2D), which is found in many tribes, particularly the Argyn (Figure 2C). The remaining haplotypes have overlapping ranges on the pCoA plot (Figures 2A,B). This indicates that there are further subclade differences among individuals within the C2-M217 haplogroup that require further subtyping. Thus, by combining the results of the pCoA and Structure analyses indicate that are broad differences among the haplogroups among the three zhuzes, in particular differences in the C2-M217, haplogroup among zhuz, as well as large differences in the frequency of certain Y-haplogroups among tribes.

## Discussion

In this study, we present the most comprehensive study of Y-STR diversity in Kazakhstan, with 1171 samples representing all of the extant tribes living within the territory of Kazakhstan. Haplotype diversity of Y-STR in Kazakhs reached a value of 0.9929, reflecting the deep paternal lineages of different origins in the sample. Our results agree with another recent study from Kazakhstan (0.9936) (Zhabagin et al., 2019), while the haplotype diversity of Kazakhs from Xinjiang (Shan et al., 2014a; Shan et al., 2014b) was found to be lower, possibly due to a founder effect of the Kazakhs that migrated to China. Most of the gene diversity (GD) estimates derived from the Y-profiler Y-STRs were consistent between Kazakhs from Kazakhstan and China (Shan et al., 2014a; Shan et al., 2014b), however, GD of DYS448 was two-fold higher in Xinjiang Kazakhs. Interestingly, the most frequent haplotype in Kazakhs from China is also one the most common haplotypes among Kazakhs from Kazakhstan. The Ht8 haplotype is associated with O2 haplogroup that accounted for 52.2% of individuals from the Naiman tribe, historically situated in Eastern Kazakhstan. Moreover, Kazakh populations in the Altai Region, Russia, are also characterized by a significant fraction of O2 individuals (31–40%) (Dulik et al., 2011; Kharkov 2012). Surprisingly, despite the high frequency of O2 haplotype in a Kazakh population studied by Shan et al. (Shan et al., 2014a; Shan et al., 2014b), no individuals with the O2 haplogroup were found in Kazakhs from Northwest China (Shou et al., 2010).

In contrast to high haplotype diversity, the overall discrimination capacity of the 15 Y-STR loci was only 0.5517. This suggests that despite including only unrelated males in the study, individuals from the same or different tribes may have identical haplotypes presumably reflecting the deep patrilineal descent among Kazakhs. For example, the discrimination capacity in Chinese Han from Shanxi Province, Northern China was 0.9865, which indicates a high potential for differentiating between male individuals in this population (Bai et al., 2013). But in Kazakh populations from Xinjiang, Northwest China the discrimination capacity was 0.5950 (Shan et al., 2014a). The discrimination capacity in our Kazakh sample is also lower compared to some data from European and Asian populations for the same set of 15 Y-STR loci (Turrina et al., 2006; Roewer et al., 2007; Robino et al., 2008; Lacau et al., 2011; Liu et al., 2020). It should be borne in mind that diversity indices vary depending on the Y-STR genotyping systems used. As the number of marker sets increases, diversity indices also increase (Purps et al., 2014; Khubrani et al., 2018; Zhabagin et al., 2019; Liu et al., 2020).

The results of the AMOVA and Mantel tests in our study confirmed that there is significantly less genetic variation among zhuzes than expected under a hierarchical model of genetic structure. This suggests that the zhuz structure is not the primary influence on genetic relationships among Kazakh tribes, and that the division into zhuzes was conditional rather than socio-territorial as suggested by other authors (Ashirbekov et al., 2018; Zhabagin et al., 2018). Nevertheless, approximately, 10% of the genetic variation among individuals was accounted for by variation among zhuzes. Furthermore, the MDS analyses indicated some differences in haplogroup structure among tribes/zhuzes, particularly for the C2-M217 haplogroup (*see* below). In the AMOVA analyses, partitioning the genetic variance within and among tribes (model: ∼tribe), revealed that ∼27% of the genetic variance is found between tribes and ∼73% within tribes. This is similar to a recent study by Ashirbekov et al. (2017) who surveyed Y-STR polymorphism, including more detailed analyses of haplogroup subclades based on SNP polymorphism, for 1269 Kazakh men sampled from 10 tribes in Southern Kazakhstan (Ashirbekov et al., 2017). Overall, they find ∼22% of the genetic variance between tribes and 78% within tribes. We did not find evidence of a relationship between genetic and geographic (birthplace) distances of individuals, despite the apparent higher frequency of the C-M217 haplogroup along the southern and western borders of Kazakhstan. We suggest that a more sophisticated spatial analysis is required to show that the C haplogroup exhibits a higher frequency in the southern part of the country.

The MDS and Structure analysis showed that there is considerable diversity in some haplogroups. Particularly interesting is the haplogroup C-M217. It is the most common haplogroup in modern Kazakhs, but the analysis shown here reveal that there at least three distinct sub-clusters of this haplogroup. Оne of the C2-M217 subgroups is dominant in tribes of the Junior zhuz (mostly in tribes Baiuly, Alimuly), one in the middle (Kongyrat tribe) and one in the Senior zhuz (in almost all tribes). We assume that these clusters represent different daughter branches of the C-M217 haplogroup. A Y-STR study by Ashirbekov et al. sampled 564 individuals (Ashirbekov et al., 2018) from ten tribes in the Senior zhuz, five tribes in the Middle zhuz and three tribes in Junior zhuz, and identified three daughter branches of the haplogroup C-M217: C-M401, C-M86 and C-M407. The authors note the predominance of the С-М401 subgroup in the tribes of the Senior zhuz, the С-М86 subgroup in the Junior zhuz tribes, and the С-М407 subgroup in the tribe of the Middle zhuz - the Kongyrat. This result is consistent with our observation. Further in-depth analysis of a number of single nucleotide markers of the C-M217 haplogroup will make it possible to determine which subgroups of this haplogroup are precisely present in the population of modern Kazakhs.

In conclusion, although several papers have described genetic polymorphism at Y-STR’s among Kazakh tribes, this is the largest study, published in English, and represents individuals from tribes in all three zhuzes as well as individuals from the Kozha and Tore tribes. Overall, we find evidence of genetic differentiation between zhuz (∼10%) and between tribes within zhuz (∼20%) suggesting that there are differences in haplogroup structure among Kazakh tribes. Although we did not find evidence of a linear relationship between genetic and geographic distance among paired individuals (i.e. non-significant Mantel test), we observed the imprint of higher frequencies of C2 haplogroups along the southern and western border of Kazakhstan, which corresponds to both the path of the Mongolian invasion and the approximate route of the ancient Silk Road. A broader spatial and temporal analysis of the Y-STR diversity among Kazakh tribes within the context of other groups in Central Asia is needed to further elucidate this dynamic history Abilev et al., 2012, Akerov, 2016, Artykbaev, 2020, Balaganskaya et al., 2011, Balanovsky et al., 2015, Barinova, 2016, Beisenov et al., 2015, Cai et al., 2011, Damba et al., 2018, Derenko et al., 2007, Ding et al., 2020, Herrera and Garcia-Bertrand, 2018, Hollard et al., 2014, Ilumä e et al., 2016, Ismagulov, 1970, Ismagulov, 1982, Jeong et al., 2019, Keyser et al., 2009, Kozhanuly, 2018, Malyarchuk et al., 2010, Meirmans, 2006, Roewer et al., 2013, Sabitov, 2013, Shi et al., 2005, Shi et al., 2013, Wei et al., 2018, Huang et al., 2018, Zegura et al., 2004, Zhabagin et al., 2016, Zhabagin et al., 2014, Zhabagin et al., 2020, Zhong et al., 2010.

## Data Availability

The raw Y-STR data were submitted to the Y-Chromosome Haplotype Reference Database (YHRD) under the accession number YA004686. Other datasets for this study can be found in the Supplementary Material.
