# Supplementary material for: Genetic Relationship Among the Kazakh People Based on Y-STR Markers Reveals Evidence of Genetic Variation Among Tribes and Zhuz
Source: Front Genet. 2022 Jan 7;12:801295. doi: 10.3389/fgene.2021.801295 (PMC8777105; doi:10.3389/fgene.2021.801295)
Supplement: Supplementary file 1 [file DataSheet1.PDF]

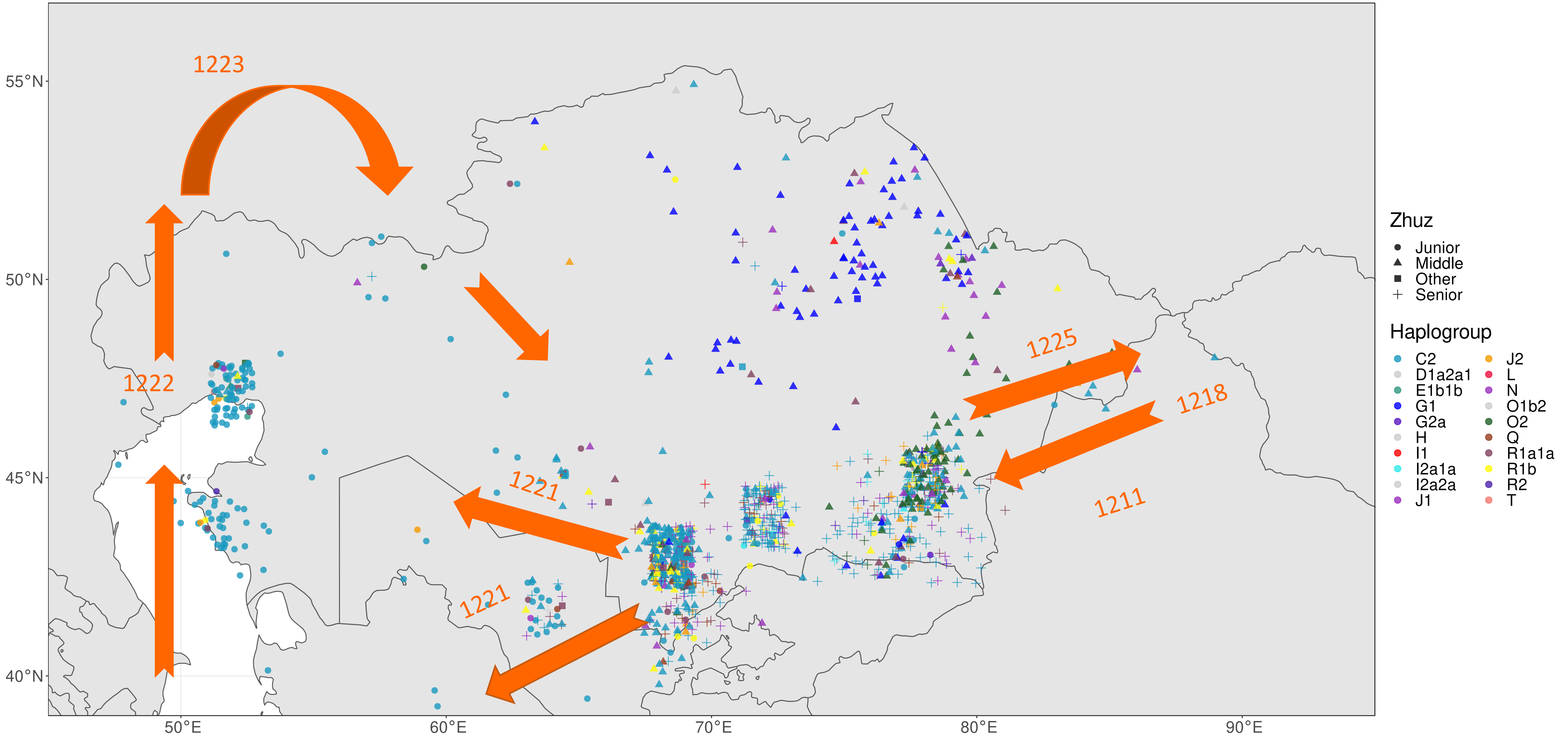

Supplementary Fig. S1. Birth location and Y-STR haplogroup of 1171 men included in this study
Estimated dates and route of Mongul invaders in the 13th century are shown in orange.

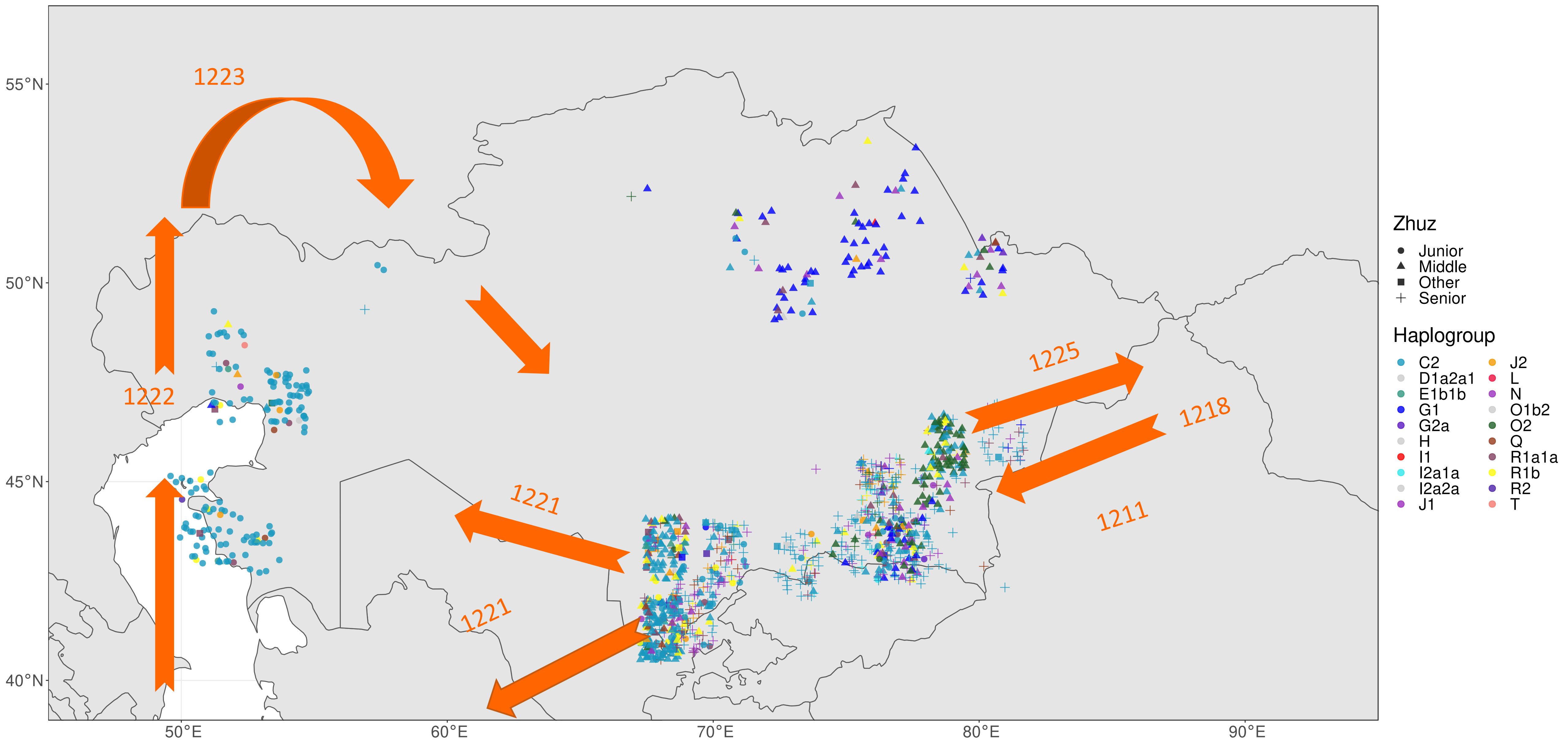

Supplementary Fig. S2. Sampling location and Y-STR haplogroup of 1171 men included in this study Estimated dates and route of Mongul invaders in the 13th century are shown in orange.

Supplementary Fig. S3 Haplogroup proportion (expressed as percent) by Kazkah tribe

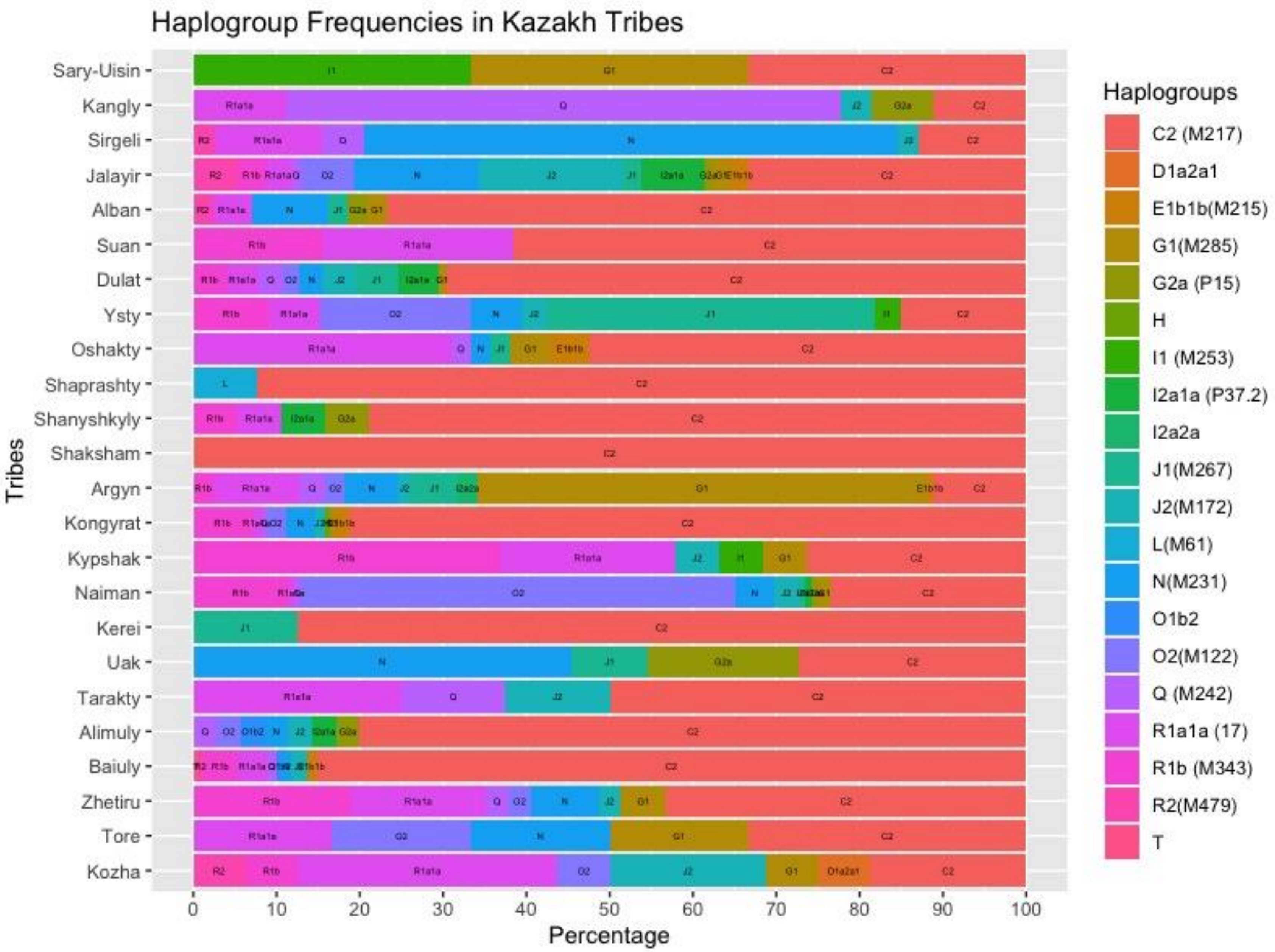

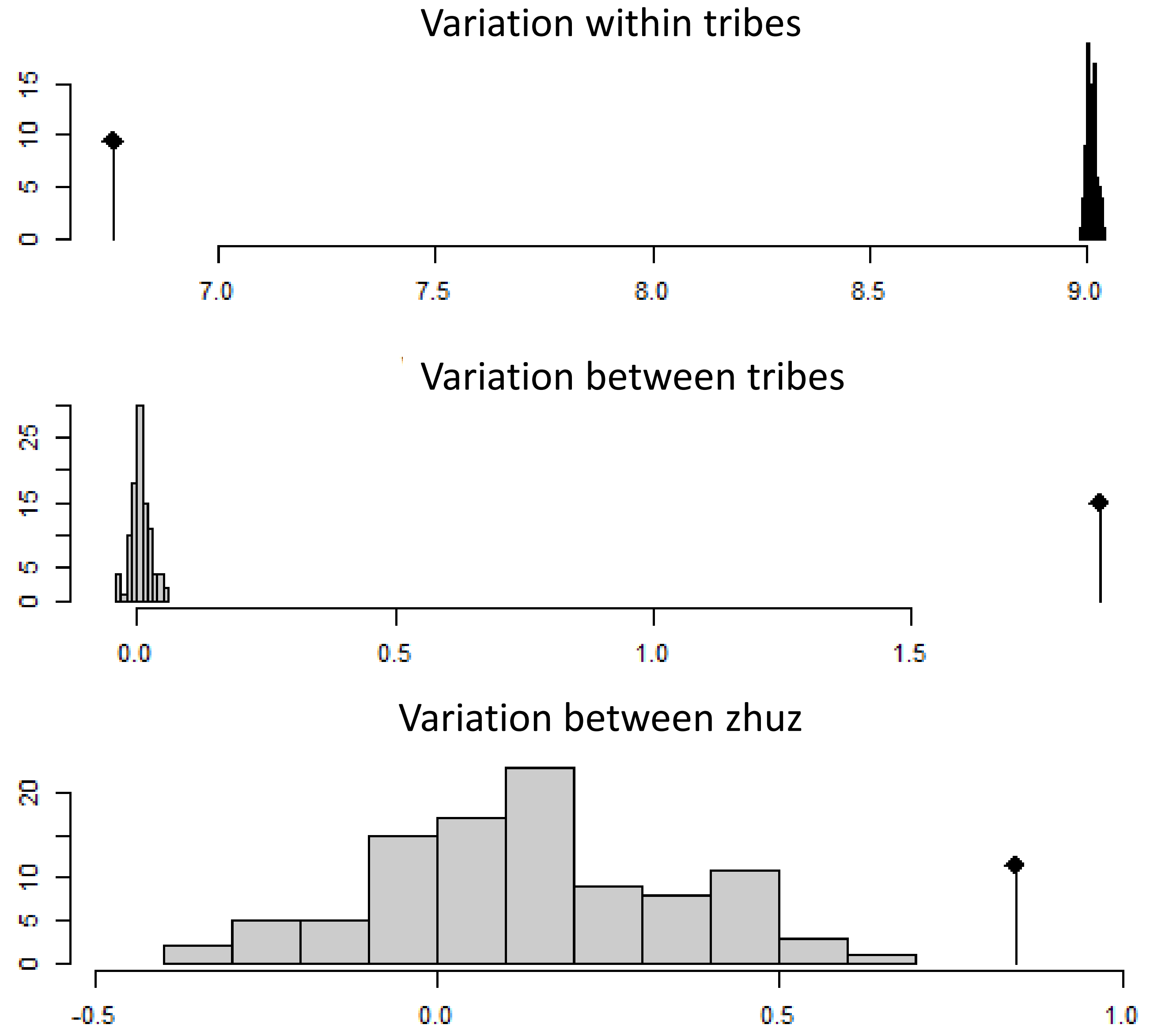

Supplementary Fig. S4. Results of the monte-carlo permutation test examining whether there is significantly more or Less variation within or between tribes, or between zhus. Lines with diamond on the top represented expectation under no structure

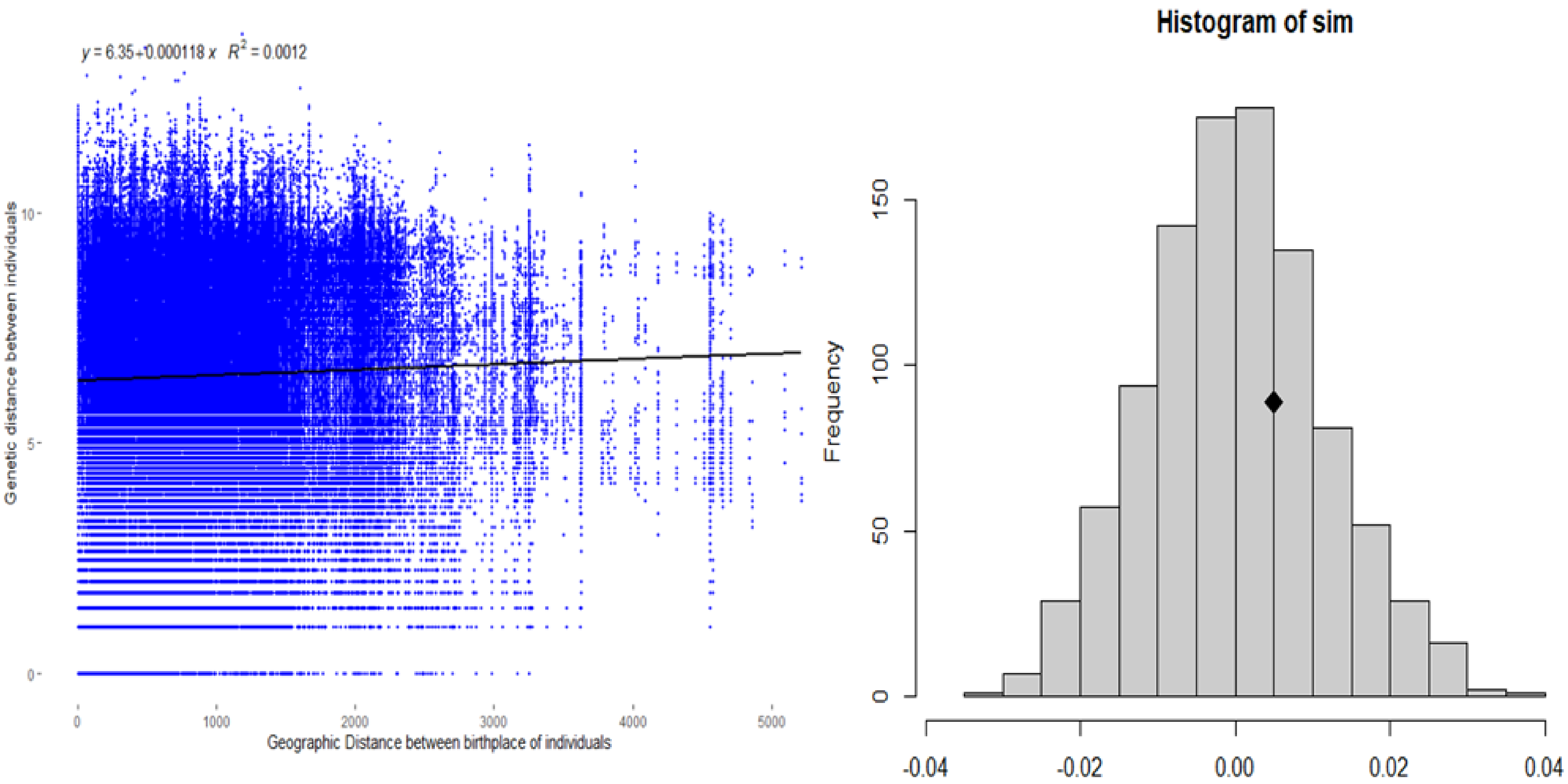

Supplementary Fig. S5. Results of the Mantel test to test for a relationship between the geographic distance between the birthplace of individuals against the genetic distance of the same two individuals (left). Results of a permutation simulation to examine whether the observed distance between geographic-genetic distances is greater than expected by chance alone (right).
